# Influence of the combined effect of desensitizing dentifrices and universal adhesives on dentin bond strength under erosive conditions

**DOI:** 10.1590/1678-7757-2023-0224

**Published:** 2023-10-27

**Authors:** Fabiana Tavares Lunardi PALHARI, Laís da Mata ALMEIDA, Priscila Christiane Suzy LIPORONI, Leandro Augusto HILGERT, Rayssa Ferreira ZANATTA

**Affiliations:** 1 Universidade de Taubaté Departamento de Odontologia Taubaté SP Brasil Universidade de Taubaté (UNITAU), Departamento de Odontologia , Taubaté , SP , Brasil .; 2 Universidade de Brasília Faculdade de Ciências da Saúde Departamento de Odontologia Brasilia DF Brasil Universidade de Brasília (UnB), Faculdade de Ciências da Saúde , Departamento de Odontologia , Brasilia , DF , Brasil .

**Keywords:** Dentin adhesives, Composite resins, Dentin, Dentin desensitizers, Dentin hypersensitivity

## Abstract

**Objective:**

This study aimed to evaluate whether the use of desensitizing dentifrices containing obliterating agents can affect bond strength of eroded/abraded dentin.

**Methodology:**

A total of 100 dentin samples were obtained from human molars. The teeth were cut into 3 mm-thickness discs and allocated in five groups (n=20), according to the toothpaste used: WoF – abrasion with fluoride-free toothpaste (Cocoricó); Arg – toothpaste containing arginine (Colgate Sensitive Pro-Relief); Nov – calcium sodium phosphosilicate toothpaste (Sensodyne Repair and Protect); SnF – fluoride-containing toothpaste (AmF/SnCl2/SnF2 – Elmex Erosion); and Control (no erosive/abrasive process). The erosive/abrasive cycle consisted of immersion in citric acid (1%, pH 2.6, 5 min, 4×/day) and abrasion (2×/day, 120–20 sec abrasion, 100 sec immersion) with each toothpaste. During intervals, samples were immersed in artificial saliva. This cycle was performed for five days. Two resin cylinders (2 mm in diameter) were constructed on each sample for the shear bond strength test using a universal adhesive system. The self-etch and etch-and-rinse (Scotchbond Universal) strategies were employed, each in half of the total sample (n=10). Bond strength (MPa) was measured in a shear test and failure modes were assessed with a stereomicroscope. Statistical analysis was performed using the two-way analysis of variance (ANOVA) and Tukey tests (p<0.05).

**Results:**

A statistically significant difference was found between the adhesive strategies tested (p<0.001), with the self-etching form showing higher values than the etch-and-rinse. Moreover, no significant differences were observed between the tested toothpastes (p=0.750) and interactions (p=0.438).

**Conclusion:**

The use of toothpaste containing obliterating agents does not affect bond strength to dentin subjected to erosive/abrasive conditions when a universal adhesive is used. However, the self-etch strategy might be preferred for eroded/abraded dentin.

## Introduction

Erosive tooth wear has been a growing concern due to modern lifestyle changes and increasing acidic food and beverages consumption, mainly by adolescents and young adults. ^1^ Recurrent erosive events associated with mechanical and physical processes, such as attrition and abrasion, lead to enamel loss and eventual exposure of dentin tubules. ^2^ In some cases, restorative and adhesive procedures are necessary to recover function and/or aesthetics and prevent dentin hypersensitivity (DH). ^3^

DH is a frequent finding in dental practice, with an average prevalence of 33% in young adults ^4^ and a concerning rate of 20% among adolescents aged from 12 to 20 years. ^5^ It is characterized by a stimulated, transient, short, and sharp pain in vital teeth presenting exposed dentin tubules in the oral environment, ^6^ with significant impairment in patients’ quality of life and daily oral activities, such as drinking, eating, speaking, and toothbrushing. ^7^ DH’s predominant risk factors are gingival recession (GR) and non-carious cervical lesions (NCCLs) ^4^ ; however, thin and cracked cervical enamel can also be related to this symptom. Still, erosive diet, lifestyle, abusive use of highly abrasive dentifrices, bruxism, and mental and sleep disorders are emerging etiological factors ^8 - 11^ The clinical management of DH is challenging and based on identifying and controlling the main etiological factors and pain using desensitizing agents with neural or obliterative approaches. ^6 , 9^

Desensitizing therapy uses professionally applied or self-applied products, including desensitizing dentifrices. They are sold as over-the-counter products and are attractive to patients presenting painful symptoms, mainly due to their easy access and low cost compared to professional treatment. ^12^ They usually contain obliterating substances such as stannous, arginine, calcium, and sodium phosphosilicate, which promote the physical sealing of the dentinal tubules by precipitation, being resistant to normal pulpal pressure and acid challenges. ^13^ Although formulations containing arginine, potassium, and stannous appear to result in significant improvement of DH symptoms, ^14^ a dentist should always indicate and supervise the use of these over-the-counter products since their indiscriminate use can delay the correct diagnosis and treatment of the disease, leading to its evolution. Still, in some cases, such as for NCCLs, the complete remission of the symptoms is usually not solved solely by dentifrices, and restorative procedures might be better indicated to restore form and function and cease lesion evolution.

Studies have investigated the influence of desensitizing therapy with dentifrices on dentin bond strength. ^15 , 16^ It is speculated that the bioactive components of the desensitizing dentifrices, such as arginine/calcium carbonate and calcium and sodium phosphosilicate, might influence the surface tension and free energy, improving the adhesive wettability. ^17^ Moreover, the presence of minerals in these dentifrices can help form a mineral-richer surface that enhances the hybrid layer formation, mainly when MDP-containing adhesives are used; however, this remains controversial. ^15 - 17^ Still, adhesive procedures performed on eroded enamel present satisfactory mechanical properties; ^18^ however, the adhesion on eroded dentin is still questionable since it is speculated that the collagen exposed during erosive events may be inadequately infiltrated by resinous monomers, creating sites more susceptible to degradation over time. ^19^

This study aimed to evaluate whether the use of desensitizing dentifrices containing obliterating agents affect the bond strength of eroded/abraded dentin. The null hypothesis tested was that the previous use of dentifrices with obliterative agents would not affect eroded dentin’s bond strength.

## Methodology

One hundred caries-free human molars extracted for therapeutic reasons were obtained from the University of Taubate Dental Clinics after approval of the local Research Ethics Committee (Protocol CAAE 28591819.0.0000.5501). Teeth were cleaned using periodontal curettes and pumice paste, then stored in deionized water at 4°C.

Dentin discs were obtained from all teeth, which were fixed in a holder with dental wax and placed at a low speed cutting machine (Isomet 1000, Buehler, Illinois, USA). They were divided into two horizontal sections: one parallel to the occlusal surface to expose dentin, and the other 1 mm below the cementoenamel junction to separate the crowns from the roots. Then, the dentin discs were standardized with 3 mm thickness from the highest pulp horn by wearing the dentin surface down with silicon carbide paper (#300 grit) at a polishing machine (Aropol E, Arotec, São Paulo, Brazil) under running water. The dentin discs were embedded in a PVC tube with acrylic resin (Resina Auto, TDV, Santa Catarina, Brazil) and polished with #600 silicon carbide paper for 60 sec to standardize the smear layer.

**Figure 1:**
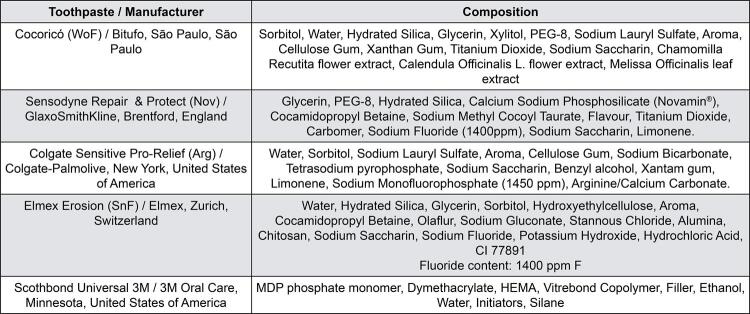
Composition of the products used in the study

The samples were randomly allocated into groups (n=20) according to the tested dentifrice: 1. WoF – Dentifrice without fluoride (Cocoricó, Bitufo); 2. Nov – Dentifrice with Novamin ^®^ bioglass (Repair & Protect, Sensodyne); 3. Arg – Dentifrice with arginine (Colgate Sensitive Pro-Relief); 4. SnF – Dentifrice with fluoride and stannous (Elmex Erosion); 5. Cont – no dentifrice used (Sound dentin). Figure 1 shows the composition of the tested products. To simulate the condition of exposure of the dentinal tubules, the samples of all groups, except the control (sound dentin), were immersed in EDTA 17.5% for 5 min. ^20^

### Erosive/abrasive cycles

All samples were submitted to erosive cycles with a citric acid solution (1%, pH=2.6) for 5 min, 4×/day, for five days. Concomitant, abrasive processes were performed 2×/day using an electric brush (Techline, EDA-01, São Paulo, Brazil) and a slurry of the respective dentifrice of each group (1:3 ratio dentifrice/artificial saliva). The specimens were immersed in the slurry and the abrasion was performed for 20 sec with a 30 g load, with back-and-forth movements from the electric brush positioned over the specimen with a grip. The load was controlled with a standard weight placed over the brush head. After 20 sec of abrasion, the samples were kept immersed in the slurry for 100 sec, then removed and washed with distilled water. ^21^ Abrasion was performed after the first and last erosive cycle of each day. The samples were stored in artificial saliva for remineralization between the erosive and abrasive procedures. The artificial saliva formula used was the one proposed by Klimek et al., and the components were: C _6_ H _8_ O _6_ , C _6_ H _12_ O _6_ , NaCl, CaCl _2_ , NH _4_ Cl, KCl, NaSCN, KH _2_ PO _4_ , CH _4_ N _2_ O, NaHPO _4_ , mucin, and deionized water. ^22^

### Restorative procedures and bond strength evaluation

After the erosive/abrasive cycles, specimens from each group were subdivided into two groups (n=10) according to the adhesive strategy tested: self-etch (SE) or etch-and-rinse (ER). A universal adhesive was used in all groups (Scotchbond Universal, 3M ESPE, St Paul, USA). For the SE groups, the adhesive was applied actively on dentin for 20 sec, followed by an air blast to evaporate the solvent; then, it was light cured with a polywave LED (1.200 mW / cm ^2^ – Bluephase, Ivoclar Vivadent, Schaan, Liechtenstein) For the ER groups, the dentin was etched with 37% phosphoric acid (Condac 37%, FGM, Santa Catarina, Brazil) for 15 sec, then rinsed for 15 sec with distilled water, dried with sterile absorbent paper, and bonded with an adhesive as described above.

For the shear bond strength evaluation, cylinders of flowable composite resin (Filtek Z350 XT flow, 3M ESPE, St Paul, USA) were built in each bonded dentin. The cylinders were built with the help of silicone tubes with a 2 mm internal diameter and 2 mm height. Two resin cylinders were built on each sample, with a minimal distance of 1 mm from each other. The samples were placed inside an opaque plastic container, with relative humidity, and stored at 37°C for seven days for a complete cure.

All samples were subjected to a shear bond strength test in a universal testing machine (mBio, BioPDI, São Carlos, Brazil). The specimens were positioned in the equipment, and a compressive force was applied parallel to the adhesive interface at a speed of 0.5 mm/min until the system failed. Shear bond strength (BS) was measured in megapascals (MPa) and obtained considering the formula: BS=F/A, in which F is the force (N) at the moment of failure, and A is the area of the adhesive interface in mm ^2^ . The BS of each sample was defined by the mean of the two cylinders measured.

### Failure pattern

After the shear test, all samples were classified by the failure pattern with a stereoscopic microscope at 20× magnification as Adhesive fracture (Ad); Cohesive in dentin failure (CD); Cohesive in resin fracture (CR); and Mixed fracture (Mix).

### Statistical analysis

Data were analyzed using the mean and standard deviation of the BS values in MPa for all groups. The samples with pre-testing (PTF) failures were disregarded for statistical purposes. Statistical analysis was performed using the two-way ANOVA test, considering the dentifrice and the adhesive strategy, followed by the post-hoc Tukey test, with significance of p<0.05. The analyses were conducted using the Jamovi software program, version 1.8 (The Jamovi Project, 2021).

## Results

The results of the two-way ANOVA test revealed a significant difference between the adhesive strategies used (p<0.001), with the self-etch technique presenting higher bond strength mean values than the etch-and-rinse. No significant difference was found between the dentifrices used (p=0.750) and between the interaction of the factors (dentifrices and adhesive strategy; p=0.438). Table 1 shows the mean values of bond strength and the results of the Tukey test.

**Table 1:** Mean (standard deviation) of bond strength (MPa) found among the group tested

	Self-etch			Etch-and-rinse			Treatment factor
	Mean	SD	PTF*	Mean	SD	PTF*	
Cont (sound dentin)	17.7	(±5.4)	0 (20)	11.2	(±4.3)	1 (19)	14.5 (±5.8)A
WoF	17.5	(±4.2)	3 (17)	12.5	(±5.9)	2 (18)	14.7 (±5.7)A
Arg	15.7	(±5.2)	0 (20)	12.7	(±6.1)	4 (16)	14.4 (±5.6)A
Nov	18.1	(±6.0)	0 (20)	10.2	(±4.5)	5 (15)	14.8 (±6.6)A
SnF	17.2	(±6.1)	0 (20)	15.4	(±5.6)	3 (17)	16.3 (±5.8)A
Adhesive strategy fator	17.2 (±5.3) A			12.5 (±5.4) B			

**Figure 2:**
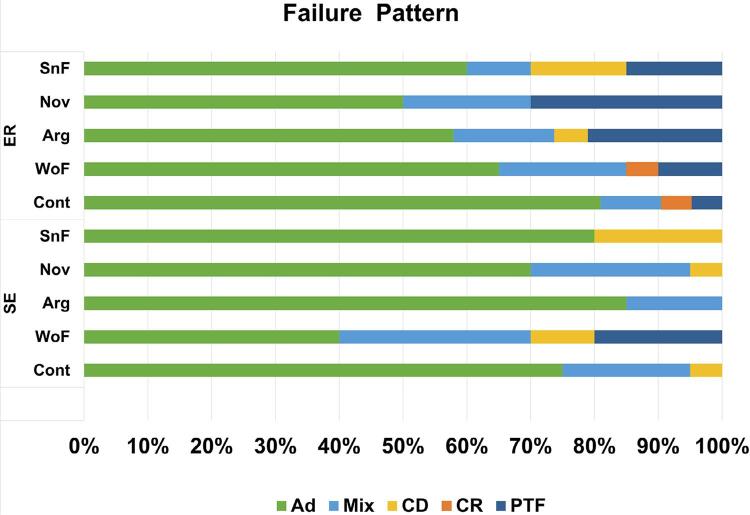
Failure pattern distribution. (Ad) Adhesive failure; (Mix) Mixed fracture; (CD) Cohesive in dentin failure; (CR) Cohesive in resin fracture; (PTF) Pre-testing failure; SE: self-etch strategy; ER: Etch-and-rinse strategy

Figure 2 presents the graph of the failure pattern analysis, which was made qualitatively . The most prevalent failure was the adhesive one, followed by mixed. The groups treated with the ER technique showed higher pre-test failures (PTF) than the SE approach.

## Discussion

Erosive events can change mineral and organic phases of dentin, and their consequence in adhesive procedures is still controversial. ^18 , 19 , 23 , 24^ Regarding the tested dentifrices, this study found no differences between the eroded/abraded dentin (WoF group) and the control group (sound dentin). In enamel, the erosive acid removes minerals from the structure, deepening into the interprismatic space, layer by layer, until complete loss of structure. ^25^ However, in dentin, demineralization exposes a mesh of collagen fibrils that slows erosion progress and can act as a protective barrier against new acidic events. ^26 , 27^ It can also resist toothbrushing abrasion, ^28^ mainly if low abrasive toothpaste and low abrasion force are used, as in this study, indicating that this might be the reason for similar results in both groups.

Regarding the tested desensitizing dentifrices, their main effect was obliterative, aiming to seal the dentin tubules by mineral precipitation. Arginine, for instance, is a positively charged amino acid naturally present in saliva that is attracted to the negatively charged exposed dentin, increasing the adhesion of calcium carbonate to the collagen fiber network, thus forming an Arg-CaCO _3_ agglomerate that enhances the calcium and phosphate ion precipitation, ^17^ and creating a mineralized plug in the dentinal tubules, reducing the flow of dentinal fluid. ^29^ The occlusive properties of the arginine-based dentifrices have been shown in literature, ^17 , 30 , 31^ and our results indicate that these agglomerates do not influence the dentin bonding strength, as similar values were found with the control group for both adhesive strategies. Moreover, regarding this toothpaste, it is essential to highlight that the presence of fluoride could not be beneficial in terms of remineralization or dentin protection since it was in MFP form, which needs saliva enzymatic breakdown to release fluoride. The calcium sodium phosphosilicate bioglass, known as Novamin ^®^ , acts by precipitating calcium and phosphate ions from saliva, forming a layer of calcium phosphate over enamel and dentin. ^32 , 33^ When in contact with saliva, the sodium ions in the Novamin glass are released, increasing the surface pH, allowing calcium and phosphate to be continuously released and precipitated over dentin, occluding the tubules. ^17^ The tested dentifrice with sodium fluoride and stannous chloride presents polyvalent fluorinated compounds, which, in addition to the formation of CaF _2_ globules, interact with the eroded tooth surface and form a mineral layer that is more acid-resistant. ^34 , 35^ It was expected that using all these dentifrices could improve remineralization and/or the formation of plugs in the entrance of the dentin tubules, decreasing the fluid flow and increasing the mineral content on the surface, which might improve adhesion. Still, it is speculated that their use could increase the dentin surface energy and wettability, favoring adhesion, ^17^ but this was not verified in our study since similar bond strength was found between the control group and the treated groups. Clinically, this might indicate that, even if the patient is under chronic use of these products for controlling dentin hypersensitivity, a restorative procedure might be performed without risks. It is important to note that pulpal pressure was not evaluated, and this could be a limitation in this study since it is associated with bond strength.

Considering the adhesive strategy, the self-etch (SE) promoted higher values than the etch-and-rinse (ER) ( Table 1 ), which might be related to the 10-MDP-containing adhesive used. The mild decalcification of the eroded dentin promoted by the adhesive in the SE strategy leads to the calcium release from the dentin surface and formation of stable self-assembled MDP-Ca salts in the form of nano layering, thus providing a simultaneous chemical and micromechanical adhesion. ^36 , 37^ Still, the possible mineral deposition induced by the desensitizing dentifrices might have favored the interaction of the 10-MDP with the dentin surface, which could have enhanced the chemical bonding of the adhesive applied. Moreover, a tendency towards not using acid etching in dentin has been noted due to the negative consequences related to exposure of the vulnerable collagen, which can collapse and impair the monomer infiltration and retention of the restoration. ^38^ In the ER strategy groups, a higher frequency of pre-testing failures was noted, indicating that the adhesion was impaired and that the possible benefits caused by the mineral precipitation of the desensitizing dentifrices could have been nullified by the use of phosphoric acid.

Therefore, this *in vitro* study indicated that the previous use of dentifrices containing obliterative agents for dentin hypersensitivity does not positively or negatively influence dentin bond strength. The remineralization promoted by the dentifrices does not interfere with the adhesion, and the adhesive strategy is more relevant for previously eroded/abraded dentin. As a limitation of this study, we highlight that artificial saliva was used during the erosive/abrasive protocol, which might reduce the remineralization promoted by fluorides. Moreover, only immediate bond strength was assessed. Therefore, we recommend that future analysis should be performed considering aging protocols.

## Conclusion

The use of dentifrices containing different obliterative agents before adhesive protocols does not interfere with the adhesion of eroded dentin. The self-etch strategy promoted higher bond strength when combined with universal adhesive when compared with the etch-and-rinse strategy.
